# English version of the self-administered Fabry Pain Questionnaire for adult patients

**DOI:** 10.1186/s13023-020-01580-9

**Published:** 2020-10-20

**Authors:** Ana Jovanovic, Philipp Klassen, Peter Heuschmann, Claudia Sommer, Mark Roberts, Nurcan Üçeyler

**Affiliations:** 1 The Mark Holland Metabolic Unit, Manchester, UK; 2 Institute of Clinical Epidemiology and Biometry, University of Würzburg, Würzburg, Germany; 3 Comprehensive Heart Failure Center, University of Würzburg, Würzburg, Germany; 4 Clinical Trial Center Würzburg, University Hospital Würzburg, Würzburg, Germany; 5 Department of Neurology, University of Würzburg, Josef-Schneider-Str. 11, 97080 Würzburg, Germany; 6 Würzburg Fabry Center for Interdisciplinary Therapy (FAZIT), University of Würzburg, Würzburg, Germany

**Keywords:** Fabry disease, Fabry-associated pain, Pain questionnaire, English version

## Abstract

**Background:**

Pain is an early symptom of Fabry disease (FD) and is characterized by a unique phenotype with mainly episodic acral and triggerable burning pain. Recently, we designed and validated the first pain questionnaire for adult FD patients in an interview and a self-administered version in German: the Würzburg Fabry Pain Questionnaire (FPQ). We now report the validation of the English version of the self-administered FPQ (enFPQ).

**Methods:**

After two forward–backward translations of the FPQ by native German and native English speakers, the enFPQ was applied at The Mark Holland Metabolic Unit, Manchester, UK for validation. Consecutive patients with genetically ascertained FD and current or previous FD pain underwent a face-to-face interview using the enFPQ. Two weeks later, patients filled in the self-administered enFPQ at home. The agreement between entries collected by supervised administration and self-administration of the enFPQ was assessed via Gwet’s AC1-statistics (AC1) for nominal-scaled scores and intraclass correlation coefficient (ICC) for interval-scaled elements.

**Results:**

Eighty-three FD patients underwent the face-to-face interview and 54 patients sent back a completed self-administered version of the enFPQ 2 weeks later. We found high agreement with a mean AC1-statistics of 0.725 for 55 items, and very high agreement with a mean ICC of 0.811 for 9 items.

**Conclusions:**

We provide the validated English version of the FPQ for self-administration in adult FD patients. The enFPQ collects detailed information on the individual FD pain phenotype and thus builds a solid basis for better pain classification and treatment in patients with FD.

## Background

Fabry disease (FD) is an X-linked lysosomal storage disorder based on mutations in the gene encoding α-galactosidase A (α-GAL). Deficiency in enzyme activity leads to cellular deposition of globotriaosylceramide (Gb3) with consecutive multiorgan disease [1]. Treatment options are i.v. enzyme replacement therapy [2, 3] and an oral chaperone [4] which may slow disease progression. Pain is one of the earliest symptoms of FD starting in childhood which may develop with a distinctive phenotype throughout adulthood in men and women [5]. Small fiber pathology is assumed to underlie FD-associated pain, however, the exact mechanisms linking the mutation with pain are unknown [6–10]. Standardized assessment of pain is crucial for clinical trials and for individual analgesic treatment. With its distinct phenotype [5], FD-associated pain is not reflected by the currently available pain questionnaires. Hence, we recently designed and validated the Würzburg Fabry Pain Questionnaire (FPQ) as the first pain questionnaire for adult FD patients, and provided an interview [11] and a self-administered version [12] in German. Here we report the validation of the English version of the FPQ (enFPQ) to allow pain assessment also to English speaking FD patients.

## Methods and patients

### Development of the enFPQ

Following a standard procedure [13], the German self-administered version of the FPQ [12] was forward-translated twice into an English version by two native German speakers and was then backward-translated twice into a German version by two native English speakers. Congruency was tested by comparison of the translated German version with the initial German version. The final enFPQ version was then applied in English speaking patients at The Mark Holland Metabolic Unit, Manchester, UK. We provide the final English version of our questionnaire in the supplement section (Additional file 1). The enFPQ records FD-pain phenotypes, triggers, and temporal course in childhood and adulthood, and its development during life with and without FD-specific treatment.

### Availability of data and materials

The datasets analyzed during the current study are available from the corresponding author on reasonable request.

### Study design

Between March 2018 and February 2020, consecutive FD patients underwent a face-to-face interview with the enFPQ during their regular visit at The Mark Holland Adult Inherited Metabolic Unit, Manchester, UK. Each question was read out to the patient and the reply was documented by the interviewer. Study participants were asked to fill in the enFPQ again on their own after 2 weeks and to send in their responses back by postal mail.

### Patients

Male and female native English-speaking patients ≥ 18 years were enrolled in our study, if FD was genetically ascertained and if patients had a current or past pain history. All patients were recruited at The Mark Holland Metabolic Unit, the NHS England national Lysosomal Storage Disorders referral center the North of England in Manchester, UK. We provide the individual genotype in the supplement section (Additional file 2).

### Statistical analysis

Statistical analysis was done using SPSS version 25 (IBM, Ehningen, Germany) and R version 3.6.3 (R Foundation, Vienna, Austria). The sample size was estimated in analogy to our previous study and to other studies [12, 14]. Since differences in current pain intensity between the interviews were considered to influence statistical reliability, we a priori determined that only patients with identical entries for current pain intensity at the two time points would be considered for analysis. For this purpose, identical pain intensity was defined as a deviation of ≤ 1 point on a numeric rating scale (NRS) for question 6 (current pain intensity) of the FPQ. A detailed synopsis of the statistical analysis is attached in Additional file 3.

## Results

### Study cohort

Table 1 gives a synopsis of the study cohort. The main characteristics of the current study population were comparable with those patients investigated during the validation study for the German version of the questionnaire [12]. 298 FD patients were screened for eligibility with 158 having a current or past pain history. Patients were invited to participate when attending a routine hospital appointment. Thirty patients declined to take part due to various reasons, for instance lack of time. Fourty-five patients either did not attend the appointment or had not received previously posted study information and hence were unable to make an informed decision whether to participate. Hence, 83/158 (53%) patients were enrolled. Of these patients, 54 underwent the first face-to-face interview and also completed and sent back their self-assessment 2 weeks later. Twenty-nine patients did not fill in and/or send back the enFPQ and were hence lost to follow-up. The main reason provided by patients was that they had forgotten to complete the second questionnaire 2 weeks later, and some stated lack of time to complete. We had to exclude 15 patients, since they reported different current pain intensities between the first and second assessment. The final study cohort thus consisted of 39 FD patients (23 men, 16 women; median age: 47 years, range 18–71 years). One patient reported pain exclusively in childhood; 23/39 (59%) patients reported pain in childhood and adulthood; 15/39 (38%) patients reported pain exclusively in adulthood. As for pain phenotypes, 34/39 (87%) patients had pain attacks, 32/39 (82%) had evoked pain, 27/39 (69%) had pain crises, and 21/39 (54%) patients reported permanent pain.

**Table 1:** Demographic data of the study population

Number of patients (N)	39
M, F (N)	23, 16
Median age (range)	47 (18–71)
Pain only in childhood (M/F)	1 (1/0)
Pain only in adulthood (M/F)	15 (9/6)
Pain in childhood and adulthood (M/F)	23 (13/10)

This table shows demographic data of the study population and the distribution of patients with pain in childhood and/or in adulthood

*M* male, *F* female

### Face-validity

All FPQ items were completed by 30/39 (77%) study participants, while 6/39 (15%) patients left question 13 unanswered (number of working days lost due to pain in the last year), either only in the self-administered FPQ session (n = 3) or in both sessions (n = 3). The remaining 3/39 (8%) patients did not answer the following questions: 7c (pain development during life), 7c and 13 (number of working days lost due to pain in the last year), and sub-questions about pain in childhood for questions 1–4. The overall acceptance by the patients was good and patients gave a positive feedback about the content and the format of the questions.

### Assessing inter-rater reliability for the self-administered and the face-to-face version

There was high agreement when comparing the face-to-face interview version with the self-administered version of the enFPQ for nominal scaled items with a mean of all AC1-statistics of 0.725 (range 0.286–1.000). All items of the questions 1–5 (the four major pain phenotypes and sensitivity impairment), except for question 4b (pain evoked by cold objects), showed at least good agreement with AC1-statistics ≥ 0.600 (Table 2). For question 8 (pain location), we found good agreement for all regions except for the arms and legs item (Table 2). Analyzing agreement of question 10 (last pain event), we faced the challenge that most of the patients had at least one pain event between the face-to-face interview and the self-report, and therefore answered this question referring to a different pain event. Hence, we only analyzed 10 patients with no pain event in the meantime with a good mean AC1-statistics result (Table 3). Question 11 (pain quality) displayed very high agreement in 7/12 items, 3/12 items showed high agreement, and 2/12 items showed adequate agreement with AC1-statistics of 0.592 and 0.578, respectively (Table 3). Results for question 12 (pain triggers) also showed high agreement except for 2 items (Table 4). Agreement of question 13 was assessed via AC1-statistics after creating a dummy variable with 3 categories (0 days, < 20 days, ≥ 20 days) and showed very high agreement (Table 4).

**Table 2:** Test–retest reliability of nominal scaled items 1–5 and 8 of the Fabry Pain Questionnaire

Questions	AC1-statistic (95% confidence interval)
1 Adulthood	0.642 (0.390–0.894)
1 Childhood	0.680 (0.494–0.866)
2 Adulthood	0.821 (0.673–0.968)
2 Childhood	0.706 (0.522–0.891)
3 Adulthood	0.815 (0.670–0.961)
3 Childhood	0.632 (0.418–0.846)
4a Adulthood	0.759 (0.588–0.930)
4a Childhood	0.618 (0.422–0.813)
4b Adulthood	0.551 (0.269–0.834)
4b Childhood	0.674 (0.486–0.861)
4c Adulthood	0.794 (0.633, 0.956)
4c Childhood	0.611 (0.411–0.810)
4d Adulthood	0.861 (0.724–0.998)
4d Childhood	0.873 (0.747–0.998)
5 Adulthood	0.772 (0.575–0.969)
5 Childhood	0.610 (0.348–0.873)
8 Hands	0.740 (0.530–0.949)
8 Feet	0.814 (0.638–0.990)
8 Back/neck	0.620 (0.359–0.880)
8 Knees	0.663 (0.416–0.910)
8 Shoulders	0.772 (0.575–0.969)
8 Other articulations	0.822 (0.654–0.991)
8 Abdomen/thorax	0.888 (0.754–1)
8 Head/jaws	0.822 (0.654–0.991)
8 Arms/legs	0.436 (0.140–0.731)

This table shows AC1-statistics of nominal scaled items 1–5 and 8 of the Fabry Pain Questionnaire. Questions 1–5 were asked for pain in childhood and adulthood, thus AC1-statistics are shown for both time periods. Information for question 8 (pain location) was collected with a diagram and AC1-statistics were calculated for each bodypart

**Table 3:** Test–retest reliability of nominal scaled items 10 and 11 of the Fabry Pain Questionnaire

Questions	AC1-statistic (95% confidence interval)
10a (n = 10)	0.756 (0.316–1)
10b (n = 10)	0.576 (0.096–1)
10c (n = 10)	0.624 (0.152–1)
11 Adulthood burning	0.605 (0.339–0.871)
11 Childhood burning	0.702 (0.468–0.936)
11 Adulthood stabbing	0.578 (0.305–0.852)
11 Childhood stabbing	0.732 (0.511–0.953)
11 Adulthood pulling	0.847 (0.699–0.994)
11 Childhood pulling	0.854 (0.714–0.995)
11 Adulthood like electric shocks	0.592 (0.327–0.857)
11 Childhood like electric shocks	0.883 (0.743–1)
11 Adulthood tearing	0.868 (0.727–1)
11 Childhood tearing	0.937 (0.844–1)
11 Adulthood don’t know	1 (–)
11 Childhood don’t know	0.822 (0.654–0.991)

This table shows AC1-statistics of nominal scaled items 10 and 11 of the Fabry Pain Questionnaire. For question 11 (quality of pain) we calculated AC1-statistics for each quality of pain

**Table 4:** Test–retest reliability of nominal scaled items 12 and 13 of the Fabry Pain Questionnaire

Questions	AC1-statistic (95% confidence interval)
12 Adulthood without trigger	0.286 (− 0.030 to 0.603)
12 Childhood without trigger	0.716 (0.492–0.940)
12 Adulthood heat	0.694 (0.458–0.931)
12 Childhood heat	0.759 (0.547–0.972)
12 Adulthood cold	0.656 (0.406–0.905)
12 Childhood cold	0.672 (0.438–0.916)
12 Adulthood fever	0.697 (0.462–0.933)
12 Childhood fever	0.852 (0.682–1)
12 Adulthood physical activity	0.543 (0.266–0.820)
12 Childhood physical activity	0.754 (0.539–0.969)
12 Adulthood sports	0.603 (0.339–0.867)
12 Childhood sports	0.810 (0.620–1)
12 Adulthood don’t know	0.971 (0.910–1)
12 Childhood don’t know	0.620 (0.362–0.877)
13	0.872 (0.722–1)

This table shows test–retest reliability of nominal scaled items 12 and 13 of the Fabry Pain Questionnaire. AC1-statistics for question 12 (pain triggers) were tested for each pain trigger

Regarding the scored interval-scaled items of the questionnaire, agreement was very high with a mean of all ICC-statistics of 0.811, and with each item showing at least high agreement (range 0.623–0.990, Table 5). For question 7b (pain development under enzyme replacement therapy, ERT), all patients with no ERT were excluded from analysis with 28 patients remaining. The calculated agreement for question 7 (pain development over time) was > 0.600 for each item, thus showing high agreement (Table 5). Questions 14 (pain influence on working ability) and 15 (pain influence on leisure activities) both showed a very high agreement with ICC-statistics ≥ 0.800 (Table 5). No assessment of agreement was calculated for free text questions 2a, 3a, and 9.

**Table 5:** Test–retest reliability of interval scaled items of the Fabry Pain Questionnaire

Questions	ICC (95% confidence interval)
6^a^	0.990 (0.981–0.995)
7a Frequency	0.735 (0.491–0.862)
7a Intensity	0.623 (0.268–0.806)
7b Frequency (n = 28)	0.820 (0.610–0.917)
7b Intensity (n = 28)	0.839 (0.647–0.927)
7c Frequency	0.844 (0.697–0.920)
7c Intensity	0.726 (0.450–0.863)
14	0.893 (0.792–0.945)
15	0.829 (0.668–0.912)

^a^We only included patients into reliability analysis of all items of the questionnaire with ≤ 1 point difference on the numeric rating scale for question 6 (current pain intensity)

This table displays test–retest reliability of interval scaled items of the Fabry Pain Questionnaire

## Discussion

We provide the English version of the Würzburg Fabry Pain Questionnaire (enFPQ), the first pain questionnaire for adult patients and self-administration. This questionnaire is based on its German FPQ version for face-to-face interview [11] and self-administration [12].

Pain in FD is distinct in its phenotype and not reflected by standardized pain questionnaires [5, 15, 16]. Its episodic nature, triggerability, and diversity in characteristics challenge systematic pain recording in FD patients, who show great variety in individual pain histories. Recently a pain questionnaire was published for children with FD [17] and we provided the first pain questionnaire for adult FD patients [11, 12]. Since its publication, we have used the FPQ at our Fabry Center for Interdisciplinary Fabry Disease (FAZIT) at the University of Würzburg, Germany with great success. The FPQ allows standardized data collection on current pain and pain in childhood which helps to categorize patients FD pain and decide on the most appropriate analgesic treatment e.g. either on demand or regular basis. Also, patients` compliance in completing the self-administered FPQ is high. The major drawback of the FPQ was, however, that it was only applicable in German speaking patients. To increase its usage, we have now validated the English version of the FPQ for self-administration. The enFPQ may be used in clinical practice when treating FD in English speaking countries as well as in multi-center clinical trials for standardized pain assessment. The enFPQ also allows remote data collection on FD pain even if patients cannot travel to a Fabry Center for a face-to-face interview.

The number of patients who entered final statistical analysis was relatively low which may be a potential source of bias due to selection, however, statistical analysis gave good to very good test–retest-reliability results for the majority of the enFPQ questions.

As observed also in our previous work validating the German versions of the FPQ, some patients gave inconsistent answers at the two sessions when asked the same question. This may be the case in patients with low frequency of pain episodes who then have difficulties in remembering the intensity or duration of a distinct pain phase. Judging if changes in pain are due to medication or the natural course of the disease may also be challenging for patients. Cognitive impairment as known in FD may be another reason contributing to inconsistency in answering repetitively asked questions [18] and which may also have influenced data collection in our study.

## Conclusion

Pain in FD has a distinct phenotype and therefore requires appropriate tools for standardized assessment in clinical practice, research, and clinical trials. The enFPQ is a useful tool to assess Fabry associated pain and may improve pain management in FD patients, and whilst pain is often a common first symptom in childhood it remains a significant problem in adults.

## Supplementary information

**Additional file 1: **English version of the Würzburg Fabry Pain Questionnaire (enFPQ).

**Additional file 2: **Individual genotype of study cohort.

**Additional file 3: **Supplementary methods.

## Data Availability

Data supporting our findings is available from the corresponding author upon qualifying request.
