# Supplementary material for: English version of the self-administered Fabry Pain Questionnaire for adult patients
Source: Orphanet J Rare Dis. 2020 Oct 20;15:296. doi: 10.1186/s13023-020-01580-9 (PMC7576746; doi:10.1186/s13023-020-01580-9)
Supplement: Supplementary file 1 — Additional file 1: English version of the Würzburg Fabry Pain Questionnaire (enFPQ). [file 13023_2020_1580_MOESM1_ESM.doc]

**Würzburg Fabry Pain Questionnaire (FPQ)**

Date: ____________________________________________________

Name: ___________________________________________________

Birth date: ________________________________________________

| **Dear Patient**  **With the following questions we would like to find out if you suffer from any pain that is typical for Fabry disease. We would like to understand the nature of your pain and its impact on your daily life. Some of the following questions may not be applicable to you. Nevertheless please answer ALL questions. Please be aware that some of the questions also refer to your childhood (i.e. before the age of 18 years). Not all given examples will fit your symptoms. Please still try to imagine similar situations in your life and answer all the questions.** |
| --- |

| **1) Do you have permanent pain in adulthood or did you have permanent pain in childhood?**  Permanent pain is a pain of any intensity (mild to unbearable) that is present for 24 hours or during most of the day. | | |
| --- | --- | --- |
|  | **In adulthood** | **In childhood** |
| Yes | O | O |
| No | O | O |
| I don`t know | O | O |

| **2) Do you have pain attacks in adulthood or did you have pain attacks in childhood?**  A pain attack is a pain of any intensity (mild to unbearable) that starts suddenly with or without a trigger, remains for a certain time period, and then disappears. | | |
| --- | --- | --- |
|  | **In adulthood** | **In childhood** |
| Yes | O | O |
| No | O | O |
| I don`t know | O | O |

| **2a) If you have pain attacks in adulthood or if you had pain attacks in childhood: how often did / how often do these pain attacks occur and for how long did / how long do these pain attacks last?** | | |
| --- | --- | --- |
|  | **In adulthood** | **In childhood** |
| Frequency  (e.g. twice a month) |  |  |
| Duration  (e.g. 1 hour) |  |  |
| I don`t know | O | O |

| **3) Do you have pain crises in adulthood or did you have pain crises in childhood?**  Pain crises are massive pain attacks that affect either parts of the body or the entire body and can last for several days (Example: Massive pain during feverous infection). | | |
| --- | --- | --- |
|  | **In adulthood** | **In childhood** |
| Yes | O | O |
| No | O | O |
| I don`t know | O | O |

| **3a) If you have pain crises in adulthood or if you had pain crises in childhood: how frequent were/are these pain crises and how long did/do they last in average?** | | |
| --- | --- | --- |
|  | **In adulthood** | **In childhood** |
| Frequency  (e.g. twice a month) |  |  |
| Duration  (e.g. 1 hour |  |  |
| I don`t know | O | O |

| **4a) Do you have in adulthood or did you have in childhood pain that could be triggered by touch?**  (Example: By walking barefoot on tiles) | | |
| --- | --- | --- |
|  | **In adulthood** | **In childhood** |
| Yes | O | O |
| No | O | O |
| I don`t know | O | O |

| **4b) Do you have in adulthood or did you have in childhood pain that could be triggered by a cold object?**  (Example: When you touch the cold steering wheel in winter) | | |
| --- | --- | --- |
|  | **In adulthood** | **In childhood** |
| Yes | O | O |
| No | O | O |
| I don`t know | O | O |

| **4c) Do you have in adulthood or did you have in childhood pain that could be triggered by a warm object?**  (Example: When you take out a warm plate from the dishwasher) | | |
| --- | --- | --- |
|  | **In adulthood** | **In childhood** |
| Yes | O | O |
| No | O | O |
| I don`t know | O | O |

| **4d) Do you have in adulthood or did you have in childhood pain that could be triggered by pressure?**  (Example: Pain that starts when you wear a narrow shoe and that disappears when you take off the shoe) | | |
| --- | --- | --- |
|  | **In adulthood** | **In childhood** |
| Yes | O | O |
| No | O | O |
| I don`t know | O | O |

| **5) Do you have in adulthood or did you have in childhood sensory impairment like numbness or tingling in the painful body area?**  (numbness = feeling that one has when e.g. one foot goes to sleep;  tingling = feeling like many little needles, when the foot awakes) | | |
| --- | --- | --- |
|  | **In adulthood** | **In childhood** |
| No | O | O |
| Numbness | O | O |
| Tingling | O | O |
| I don`t know | O | O |

| **6) What is your pain intensity at the moment? Zero means “no pain” and ten means “worst pain imaginable”.** | |
| --- | --- |
|  | **Please indicate only one number** |
| 0 – 1 – 2 – 3 – 4 – 5 – 6 – 7 – 8 – 9 – 10  No Worst pain imaginable  pain | |

| **7) How did your pain develop over time (with or without treatment)?** | |
| --- | --- |
| **a) since last visit here**  Please indicate the number that is most suitable for you. The scales run from -10 to 10. With regard to “frequency” -10 means that you suffer from pain less frequently; zero means that nothing has changed; 10 means that you suffer from pain much more frequently. | |
|  | **Please indicate only one number** |
| **WITH REGARD TO FREQUENCY:**  -10 -9 -8 -7 -6 -5 -4 -3 -2 -1 0 1 2 3 4 5 6 7 8 9 10  Pain less frequent than before  Pain more frequent then before    **WITH REGARD TO INTENSITY:**  -10 -9 -8 -7 -6 -5 -4 -3 -2 -1 0 1 2 3 4 5 6 7 8 9 10  Pain less intensive than before  Pain more intensive than before  I cannot answer this question: **O** | |
| **b) under enzyme replacement therapy**  Please indicate the number that is most suitable for you. The scales run from -10 to 10. With regard to “frequency” -10 means that you suffer from pain less frequently; zero means that nothing has changed; 10 means that you suffer from pain much more frequently. (If you do not receive enzyme replacement therapy please indicate “I cannot answer this question”). | |
|  | **Please indicate only one number** |
| **WITH REGARD TO FREQUENCY:**  -10 -9 -8 -7 -6 -5 -4 -3 -2 -1 0 1 2 3 4 5 6 7 8 9 10  Pain less frequent than before enzyme replacement therapy  Pain more frequent than before enzyme replacement therapy  **WITH REGARD TO INTENSITY:**  -10 -9 -8 -7 -6 -5 -4 -3 -2 -1 0 1 2 3 4 5 6 7 8 9 10  Pain less intensive than before enzyme replacement therapy  Pain more intensive than before enzyme replacement therapy  I cannot answer this question: **O** | |
| **c) during life (from birth until now)**  Please indicate the number that is most suitable for you. The scales run from -10 to 10. With regard to “frequency” -10 means that you suffer from pain less frequently; zero means that nothing has changed; 10 means that you suffer from pain much more frequently. | |
|  | **Please indicate only one number** |
| **WITH REGARD TO FREQUENCY:**  -10 -9 -8 -7 -6 -5 -4 -3 -2 -1 0 1 2 3 4 5 6 7 8 9 10  Pain less frequent than before  Pain more frequent than before  **WITH REGARD TO INTENSITY:**  -10 -9 -8 -7 -6 -5 -4 -3 -2 -1 0 1 2 3 4 5 6 7 8 9 10  Pain less intensive than before  Pain more intensive than before  I cannot answer this question: **O** | |

| **8) Please indicate the body areas that are mainly affected when you have pain.** |
| --- |


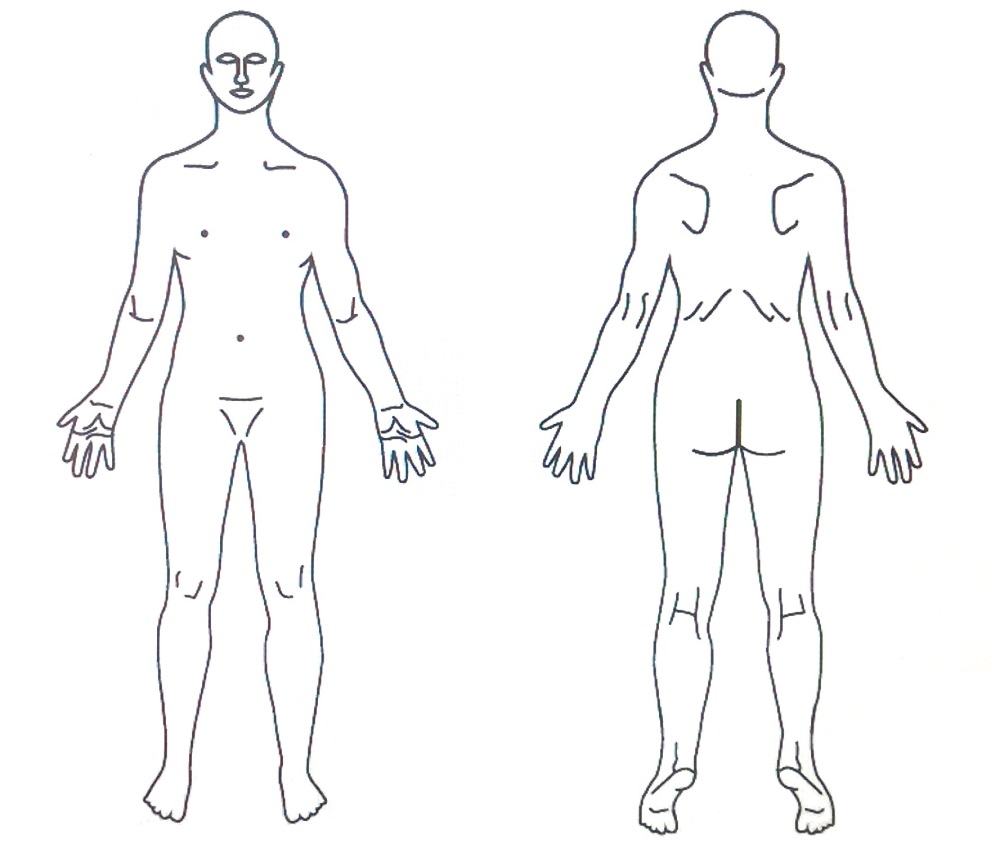


| **9) Which analgesic drugs do you take? Please indicate the generic name, the dosage and the intake regimen per day.** | |
| --- | --- |
| I don`t take analgesic drugs | O |
| I take the following drugs:   | **Drug** | **Dosage** | **Daily intake regimen** | | --- | --- | --- | | Example: Pregabalin | 75 mg | 1-0-1 | | Example: Acetaminophen | 500 mg | When required | |  |  |  | |  |  |  | |  |  |  | |  |  |  | |  |  |  | |  |  |  | |  |  |  | | |

| **10) When was the last time you had pain? Please indicate the data as precise as possible. If you cannot remember please indicate the approximate time period.** |
| --- |
|  |

| **10a) What type of pain was your last pain?** | |
| --- | --- |
|  | **You can chose several options** |
| **Permanent pain**  (Permanent pain is pain of any intensity (mild to unbearable) that is present for 24 hours or during most of the day) | O |
| **Pain attack**  (A pain attack is pain of any intensity (mild to unbearable) that starts suddenly, remains for a certain time period, and then disappears) | O |
| **Pain crisis**  (Pain crises are massive pain attacks that affect either parts of the body or the entire body and that can last for several days) | O |
| **Pain that is triggered by normally non-painful stimuli** | O |
| Other: |  |
| I don`t know | O |
| **10b) The last time you had pain: what was its maximum intensity on a scale from zero to ten? Zero means “no pain” and ten means “worst pain imaginable”.** | |
|  | **Please indicate only one number** |
| 0 – 1 – 2 – 3 – 4 – 5 – 6 – 7 – 8 – 9 – 10  No Worst pain imaginable  Pain | |

| **10c) The last time you had pain: what was its average intensity on a scale from zero to ten? Zero means “no pain” and ten means “worst pain imaginable”.** | |
| --- | --- |
|  | **Please indicate only one number** |
| 0 – 1 – 2 – 3 – 4 – 5 – 6 – 7 – 8 – 9 – 10  No Worst pain imaginable  Pain | |

| **11) How does your pain feel? You can indicate several answers.** | | |
| --- | --- | --- |
|  | **In adulthood** | **In childhood** |
| Burning | O | O |
| Stabbing | O | O |
| Drawing | O | O |
| Like electric shocks | O | O |
| Tearing | O | O |
| Other: | | |
| I don`t know | O | O |

| **12) Are there triggers for your pain? You can indicate several answers.** | | |
| --- | --- | --- |
|  | **In adulthood** | **In childhood** |
| Spontaneous pain without triggers | O | O |
| Heat | O | O |
| Cold | O | O |
| Fever | O | O |
| Physical activity | O | O |
| Sports | O | O |
| Other: | | |
| I don´t know | O | O |

| **13) How many days have you been unable to work (including housework) in the last year due to the pain? Weekends are included on which you were e.g. not able to leave your bed due to pain.** |
| --- |
| ________________ days without work |

| **14) How much does pain influence your working ability (including housework) in general on a scale from zero to ten? Zero means “no influence” and ten “working impossible”.** | |
| --- | --- |
|  | **Please indicate only one number** |
| 0 – 1 – 2 – 3 – 4 – 5 – 6 – 7 – 8 – 9 – 10  Not at all impaired Working impossible | |

| **15) How much does pain influence your leisure activities in general on a scale from zero to ten? Zero means “no influence” and ten “leisure activities are impossible”.** | |
| --- | --- |
|  | **Please indicate only one number** |
| 0 – 1 – 2 – 3 – 4 – 5 – 6 – 7 – 8 – 9 – 10  Not at all impaired Leisure activities impossible | |
