# Supplementary material for: English version of the self-administered Fabry Pain Questionnaire for adult patients
Source: Orphanet J Rare Dis. 2020 Oct 20;15:296. doi: 10.1186/s13023-020-01580-9 (PMC7576746; doi:10.1186/s13023-020-01580-9)
Supplement: Supplementary file 2 — Additional file 2: Individual genotype of study cohort. [file 13023_2020_1580_MOESM2_ESM.docx]

**Supplementary Material 2: Individual genotype of study cohort**

| **Study ID** | **Mutation** | **Classification** |
| --- | --- | --- |
| 001 | N215S | non-classical |
| 002 | P265S | classic |
| 003 | P265S | classic |
| 005 | P293S | classic |
| 008 | R112C | classic |
| 009 | c.194+1g>a | classic |
| 010 | IVS2+4delag | intronic |
| 011 | IVS2+4delag | intronic |
| 012 | C.1011-1029del19 | classic |
| 014 | D92Y | classic |
| 018 | N215S | non-classical |
| 019 | T410I | classic |
| 020 | R112C | classic |
| 021 | N215S | non-classical |
| 022 | I242F | classic |
| 024 | R112C | classic |
| 026 | IVS6-1G>C | classic |
| 027 | G361X | classic |
| 028 | N215S | non-classical |
| 030 | N215S | non-classical |
| 033 | R301X | classic |
| 034 | P259R | classic |
| 035 | R301Q | non-classical |
| 037 | N215S | non-classical |
| 040 | R100K | classic |
| 041 | R342X | classic |
| 042 | R342X | classic |
| 043 | R363C | non-classical |
| 045 | N215S | non-classical |
| 046 | IVS6-1G>A | classic |
| 047 | A257P | non-classical |
| 048 | A257P | non-classical |
| 050 | Y88D | classic |
| 051 | R118C | likely benign |
| 052 | IVS1+1G>A | classic |
| 053 | IVS1+1G>A | classic |
| 057 | IVS3+1G>C | classic |
| 059 | c.719delA | classic |
| 060 | M353R | classic |
| 062 | Y88D | classic |
| 063 | IVS1+1G>A | classic |
| 065 | G361X | classic |
| 066 | Exon 4 to 7 deletion | unknown |
| 072 | M353R | classic |
| 073 | c.107_125dup | unknown |
| 076 | N215S | non-classical |
| 077 | N215S | non-classical |
| 078 | I232T | likely classic |
| 079 | c.180delA | unknown |
| 080 | G183S | classic |
| 081 | Q321E | classic |
| 083 | c.5482?-1290+? | unknown |
| 084 | P409A | classic |
| 085 | G183S | classic |
