# Supplementary material for: English version of the self-administered Fabry Pain Questionnaire for adult patients
Source: Orphanet J Rare Dis. 2020 Oct 20;15:296. doi: 10.1186/s13023-020-01580-9 (PMC7576746; doi:10.1186/s13023-020-01580-9)
Supplement: Supplementary file 3 — Additional file 3: Supplementary methods. [file 13023_2020_1580_MOESM3_ESM.docx]

**Supplementary methods**

**Statistical analysis**

Statistical analysis was performed in analogy to the analysis in our previous study [[1](#_ENREF_1)]. For calculating the agreement of nominally scaled items of the questionnaire (questions 1-5 and 8-13), we used Gwet’s AC1-statistics as a more advanced and adequate method instead of assessing kappa-coefficients because of its known shortcomings, as described before [[2](#_ENREF_2)]. There are mainly two reasons, why Gwet’s AC1-statistics is superior to kappa-coefficients for nominally-scaled items, like in our case. First, kappa is not a particular “chance-corrected measure of agreement”, because the calculation of chance agreement is based on the assumption that rater decisions are independent, which is not given in our evaluation. Second, raters` classification probabilities and the corresponding category’s prevalence in the subject population may have a relevant influence on the kappa statistics [[2](#_ENREF_2), [3](#_ENREF_3)]. Thus, we used Gwet’s AC1-statistic to counter these weaknesses. For evaluating the agreement of scored, interval-scaled items (questions 6-7 and 14-15) of the two questionnaire assessments (method one, face-to-face enFPQ; method two, self-administered enFPQ), we calculated the inter-rater reliability via intraclass-correlation-coefficients (ICCs), as previously described [[4](#_ENREF_4)].

For AC1-statistics and ICCs between 0 and 0.2, agreement was defined as very low, between 0.2 and 0.4 as low, between 0.4 and 0.6 as adequate, between 0.6 and 0.8 as good, and between 0.8 and 1.0 as very good.

For question 10 (last pain event) only 10 patients were available (see results), hence we created categorical dummy variables with 4 categories (0=no pain, 1-3=mild pain, 4-7=intermediate pain, 8-10=severe pain) for the original NRS entries and calculated the agreement via AC1-statistics. For assessing agreement of question 8 (pain location) we created dummy variables (yes/no) to calculate agreement for each pain location via AC1-statistics and calculation of AC1-statistics for question 13 (missing working days during last year including household), was done after creating a dummy variable with 3 categories (0 days, <20 days, ≥20 days).

**References:**

1. Magg B, Riegler C, Wiedmann S, Heuschmann P, Sommer C, Üçeyler N. Self-administered version of the Fabry-associated pain questionnaire for adult patients. Orphanet J Rare Dis. 2015;10:113.

2. Gwet KL. Computing inter-rater reliability and its variance in the presence of high agreement. Br J Math Stat Psychol. 2008;61:29-48.

3. Nolte CH, Malzahn U, Rakow A, Grieve AP, Wolfe CD, Endres M et al. [The German version of the satisfaction with stroke care questionnaire (SASC) for stroke patients]. Fortschr Neurol Psychiatr. 2010;78:355-9.

4. Shrout PE, Fleiss JL. Intraclass correlations: uses in assessing rater reliability. Psychol Bull. 1979;86:420-8.
